# Temporal Expression Dynamics of Plant Biomass-Degrading Enzymes by a Synthetic Bacterial Consortium Growing on Sugarcane Bagasse

**DOI:** 10.3389/fmicb.2018.00299

**Published:** 2018-02-26

**Authors:** Diego Javier Jiménez, Maryam Chaib De Mares, Joana Falcão Salles

**Affiliations:** ^1^Microbial Ecology Cluster, Groningen Institute for Evolutionary Life Sciences, University of Groningen, Groningen, Netherlands; ^2^Department of Biological Sciences, Universidad de los Andes, Bogotá, Colombia

**Keywords:** bacterial consortium, expression dynamics, lignocellulolytic enzymes, metatranscriptomics, sugarcane bagasse, synergism

## Abstract

Plant biomass (PB) is an important source of sugars useful for biofuel production, whose degradation efficiency depends on synergistic and dynamic interactions of different enzymes. Here, using a metatranscriptomics-based approach, we explored the expression of PB-degrading enzymes in a five-species synthetic bacterial consortium during cultivation on sugarcane bagasse as a unique carbon source. By analyzing the temporal expression dynamics of a selection of enzymes we revealed the functional role of each consortium member and disentangled the potential interactions between them. Based on normalized expression values and the taxonomic affiliation of all the transcripts within thirty carbohydrate-active enzyme (CAZy) families, we observed a successional profile. For instance, endo-glucanases/-xylanases (e.g., GH8, GH10, and GH16) were significantly expressed at 12 h, whereas exo-glucanases (e.g., GH6 and GH48) and α-arabinosidases/β-xylosidases (e.g., GH43) were highly expressed at 48 h. Indeed, a significant peak of extracellular β-xylosidase activity was observed at this stage. Moreover, we observed a higher expression of several CAZy families at 12–48 h, suggesting easy access to the main plant polysaccharides. Based on this evidence, we predicted that the highest level of collaboration between strains takes place at the initial stages of growth. Here, *Paenibacillus*, *Brevundimonas*, and *Chryseobacterium* were the most important contributors, whereas *Stenotrophomonas* was highly active at the end of the culture (96–192 h) without contributing to a large extent to the expression of lignocellulolytic enzymes. Our results contribute to the understanding of enzymatic and ecological mechanisms within PB-degrading microbial consortia, yielding new perspectives to improve the PB saccharification processes.

## Introduction

Sugarcane bagasse (SCB) has been considered a useful bioenergy feedstock due to its low cost and huge availability (Cardona et al., 2010). As the majority of agricultural residues, SCB is composed of about 40% cellulose (β-1,4-linked D-glucose units), 25% hemicellulose, 20% lignin and a small percentage of pectin (Kim and Day, 2011; Szczerbowski et al., 2014). In agricultural residues, three main polysaccharides constitute the hemicellulosic fraction, i.e., xylan/arabinoxylan, xyloglucan, and galacto(gluco)mannan. These polymers are usually classified according to the sugar residues present in the backbone. For instance, xylan/arabinoxylan is composed of β-1,4-linked D-xylose units, which may be substituted by different side groups (e.g., D-galactose, L-arabinose, and glucuronic acid) (van den Brink and de Vries, 2011; de Souza, 2013). Thus, due to the high SCB complexity, an efficient release of the monosaccharides requires the synergic interaction of several enzymes (e.g., endo/exo -glucanases, endo-xylanases, β-glucosidases, α-arabinosidases, lytic polysaccharide monooxygenases, β-xylosidases, and α-glucuronidases) (Gao et al., 2011; Rytioja et al., 2014; Kim et al., 2017). In the production of biofuels, an efficient SCB saccharification process still represents a bottleneck due to the recalcitrant nature of the most plant biomass (PB) (Himmel et al., 2007; Canilha et al., 2012; Visser et al., 2015).

Recently, constructed PB-degrading microbial consortia have proven to be a excellent sources of (hemi)cellulolytic enzyme cocktails (Park et al., 2012; Jiménez et al., 2015b). A common strategy to obtain these types of consortia is by the dilution-to-stimulation method (Lee et al., 2013). In this approach, a microbial community (inoculum) is set to grow on agricultural residues in liquid batch cultures that are sequentially diluted across several transfers (Zhang et al., 2013; de Lima Brossi et al., 2015; Jiménez et al., 2016). Throughout the enrichment, the inoculum gradually changes in composition, yielding a specialized microbial consortium with progressively reduced richness. In this process, two types of successional dynamics may occur: the first one is the succession along the sequential transfers – the selection phase – and the other one occurs within the relative ‘stable’ consortia, where microbial species with complementary activities synergistically degrade the PB substrate (Jiménez et al., 2017). These PB-degrading microbial consortia are usually composed of hundreds of microbial species, from which small core sets of organisms – i.e., the minimal effective consortium – contribute to most of the degradation process whereas the remaining species could act mostly as ‘cheaters,’ consuming the sugars released by the effective core. For instance, in our previous studies, *Sphingobacterium* and *Klebsiella* species were the most relevant degraders in microbial consortia cultivated on wheat straw (Jiménez et al., 2014a, 2015a,b; Maruthamuthu et al., 2016). These types of microbial enrichments have served as sources for the isolation of efficient PB-degrading strains, which can be further used either as source of new enzymes or as active strains useful for the design of synthetic microbial communities (Evans et al., 2017).

The efficiency of PB-degrading microbial consortia relies probably on the level of complementarity and synergism that is achieved, either in terms of enzymatic pool (or the timing of enzymatic release) and/or number of active species. The temporal aspects of microbial PB degradation are of high relevance, because enzyme-enzyme synergism can occur in a temporal scale, as is the case for the conversion of cellulose to cellobiose and the subsequent release of glucose (Lynd et al., 2002). Two recent studies explored the temporal expression dynamics of lignocellulolytic enzymes in PB-degrading microbial consortia. In these, the expression profile of some glycoside hydrolases (GHs) was assessed by metaproteomics (Zhu et al., 2016) as well as through the hybridization of total consortium RNA against a new CAZychip (Abot et al., 2016). For instance, Zhu et al. (2016) have evaluated the secreted proteins in a corn stover-adapted consortium along 1, 3, and 7 days of growth. They detected the CAZy families GH1, GH3, GH9, GH10, GH11, GH13, GH43 and GH94 after 24 h of cultivation. However, an in-depth understanding of the enzymatic mechanisms and ecological interactions in such consortia is still missing. The modulation of the species diversity/composition in a synthetic microbial consortium provides an experimental tool to unravel the enzymatic mechanisms that can potentially lead to a significant improvement in PB saccharification processes (Lindemann et al., 2016; Cavaliere et al., 2017; Jiménez et al., 2017). In addition, here we posit that to understand interactions, it is crucial to disentangle the temporally explicit expression dynamics of lignocellulolytic enzymes in these microbial systems.

In this study, we analyzed the temporal expression dynamics of thirty PB-degrading enzymes in a five-species synthetic bacterial consortium – previously selected for complementarity and high degradation potential – along a single culture batch on SCB. A successional expression profile was observed to occur in the consortium, where *Paenibacillus*, *Brevundimonas*, and *Chryseobacterium* were the most relevant contributors to the expression of lignocellulolytic enzymes. Ecological interactions and successional dynamics between the consortium members were discussed based on a recently developed conceptual framework (Jiménez et al., 2017). Our metatranscriptomics analysis portrays the temporal enzymatic processes underlying a synthetic PB-degrading microbial consortium, improving our ecological understanding on these systems and giving new perspectives to improve PB saccharification processes.

## Materials and Methods

### Growth of the Synthetic Bacterial Consortium on Different Substrates

Five phylogenetically different bacterial strains isolated from a soil-derived lignocellulolytic microbial consortium were selected. This selection was based on parameters established by Puentes-Téllez and Salles (2018), after screening of 60 synthetic five-species consortia varying in their functional metabolic diversity and drawn from a pool of 18 bacterial species. The selected strains (mixture 48) when grown together on PB (after 96 hrs of cultivation), could degrade up to 50% of the total lignocellulose fraction, with specific degradation percentages of 43.01, 39.65, 69.18% of lignin, cellulose and hemi-cellulose, respectively (Puentes-Téllez and Salles, 2018). Based on previous 16S rRNA gene sequencing, these strains were identified, at the genus level, as *Stenotrophomonas*, *Paenibacillus*, *Microbacterium*, *Chryseobacterium*, and *Brevundimonas*.

In order to analyze the temporal growth dynamics of the synthetic bacterial consortium, we cultivated the bacterial mixture (five strains) on four different substrates as sole carbon source varying in their complexity (glucose -GLC, carboxymethylcellulose -CMC, xylan -XYL and sugarcane bagasse -SCB). Reagents like GLC, CMC and XYL were obtained from Sigma–Aldrich. The SCB was previously milled into small pieces (≤1 mm), washed twice with 70% of ethanol and distilled water, and then dried at 55°C for 48 h. Firstly, each bacterial strain was cultivated in 5 ml of LB broth for 48 h. The cultures were centrifuged (6,500 g × 10 min) and the cells were washed with 1 ml of 0.9% NaCl. Absorbance (OD_600 *nm*_) for all the strains was adjusted with 0.9% NaCl at 1.0 in order to ensure similar starting densities. Subsequently, 25 μl of each strain (relation 1:1) were inoculated into 25 ml of sterile mineral salt medium containing 0.5% of each substrate, vitamins and trace element solutions (Jiménez et al., 2014b). Triplicate flasks per time point were incubated at 28°C in aerobic conditions (180 rpm) and samples were taken at 12, 48, 96, and 192 h for subsequent analysis. Microbial growth was evaluated by absorbance (OD_600 *nm*_) for each time point. Absorbance in the SCB-derived cultures was measured directly at the upper layer, after substrate sedimentation. Two controls, i.e., one without substrate and the other without microbial source were set up (**Supplementary Figure S1**).

### Quantification of Sugars, Proteins and Enzymatic Activities in the Extracellular Fractions from the Synthetic Bacterial Consortium Cultivated on Xylan and Sugarcane Bagasse

Sugar and protein concentrations, as well as the enzymatic activities of the consortium cultivated on XYL and SCB were measured in the extracellular fraction. To obtain these fractions, 10 ml of each culture at each time point were centrifuged (6,500 g × 8 min). The supernatant was clarified by passing through a 0.22 μm filter. Quantity of sugars and proteins was obtained by 3,5-dinitrosalicilic acid (DNS) method (Miller, 1959) and the Quick Start^TM^ Bradford Protein Assay (Biorad, Hercules, CA, United States), respectively. Enzymatic activities related with PB degradation were evaluated using pNP-labeled substrates, i.e., *p*-nitrophenyl α-D-glucopyranoside (pNPαGlu), *p*-nitrophenyl β-D-xylopyranoside (pNPβXyl), *p*-nitrophenyl β-D-galactopyranoside (pNPβGal) and *p*-nitrophenyl α-D-mannopyranoside (pNPαMan). The reaction mixtures consisted of 200 μl of 7.5 mM of each *p*-nitrophenol-glycoside (diluted in 50 mM of Tris-HCL pH 7.5) and 150 μl of each supernatant. The mixtures were incubated at 37°C for 4 h, after which the reactions were stopped on ice. Two negative controls were used for all assays: (i) reaction mixture without pNP-substrate; (ii) reaction mixture using sterile water. Enzymatic activities were determined from the measured absorbance units using a standard calibration curve. Activity was detected by the presence of yellow color and the amount of para-nitrophenol liberated was measured by absorbance at 410 nm (Maruthamuthu et al., 2017). All the analyses were performed using three biological replicates and standard deviations (σ) were obtained.

### Extraction of Total RNA and Metatranscriptomic Sequencing

Total microbial RNA extraction from the consortium was carried out along the batch of culture on XYL and SCB. For each time point (12, 48, 96, and 192 h) three biological replicates were taken. Briefly, 10 ml of RNAprotect Bacteria Reagent (Qiagen, Hilden, Germany) were added to 5 ml of microbial culture. After 5 min (at room temperature) the mixture was centrifuged at 5,000 *g* for 10 min. The cell pellets were lysed using proteinase K (50 mg/ml) and lysozyme (15 mg/ml), and the RNA extraction was performed using the RNeasy Mini Kit (Qiagen, Hilden, Germany) following the manufacturer’s instructions. The quality and quantity of the extracted total RNA was checked by non-denaturing agarose gel electrophoresis and absorbance ratios 260/280 nm. Depletion of ribosomal RNA (using Ribo-Zero^TM^ Kit), mRNA libraries preparation and sequencing (Illumina NextSeq 2 × 150 bp; 1 lane) were performed at LGC Genomics (Berlin, Germany).

### Analysis of Metatranscriptomic Data

A total of 116,205,704 quality filtered paired-end reads (>20 bp) from 21 samples were used as input for *de novo* transcript assembly using Trinity (Haas et al., 2013). Transcript abundance estimation and normalization was performed using scripts included in the Trinity package. The script *abundance_estimates_to_matrix.pl* was used to create a matrix with the estimated RNA-Seq fragment raw counts that were used for differential expression analysis with edgeR (Robinson et al., 2010). To enable direct in-between sample comparison of transcript abundances, raw abundances were converted to TPM (transcripts per million transcripts) and TMM (trimmed mean of M-values) normalized in RSEM (RNA-Seq by Expectation Maximization) (Dillies et al., 2012). The script *analyze_diff_expr.pl* was used to extract those transcripts that were at least fourfold differentially expressed at a FDR (false discovery rate) corrected *P*-value cut off of 0.001 in any of the pairwise sample comparisons, followed by hierarchical clustering based on the Pearson correlation matrix or pairwise comparisons of samples using edgeR (Robinson et al., 2010). Moreover, total transcript contigs were converted to potential coding regions using the TransDecoder function developed within Trinity. For functional classification, the proteins were annotated using the GhostKOALA (Kanehisa et al., 2016) and dbCAN web-platforms (Yin et al., 2012). In order to unveil the relative expression dynamics of lignocellulolytic proteins, and in accordance with previous omics studies (Simmons et al., 2014; Zhou et al., 2014; Berlemont and Martiny, 2015; Jiménez et al., 2015a; 2016; Abot et al., 2016; Antunes et al., 2016; Zhu et al., 2016), we selected thirty carbohydrate-active enzyme (CAZy) families (Lombard et al., 2014) that are highly relevant in the deconstruction of PB. All transcripts affiliated within those CAZy families were manually annotated by BLASTp against the NCBI-nr database. The taxonomic affiliation of each transcript was performed by manually taking the most frequent BLASTp hit between the top-50. Finally, the TMM-normalized TPM values were added to each transcript classification and summed up to obtain the values per each CAZy family (Pfreundt et al., 2016). Relative expression of the CAZy families that segregated significantly between the time points were identified using random forest analysis with 1000 trees followed by the Boruta algorithm for feature selection (average *z*-scores of 1000 runs > 3) (Cardenas et al., 2015). All metatranscriptomic data are publically accessible on the MG-RAST (Meyer et al., 2008) server under the IDs mgm4734165.3 to mgm4734173.3; and mgm4735448.3 to mgm4735459.3.

## Results

### Growth of the Synthetic Bacterial Consortium on Different Substrates

**FIGURE 1:**
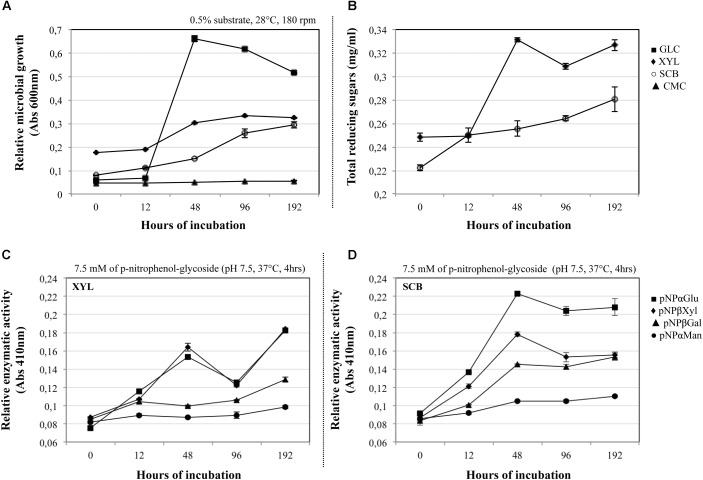
**(A)** Microbial growth (OD_600 *nm*_) of the synthetic bacterial consortium on glucose –GLC (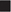), carboxymethylcellulose –CMC (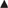), xylan -XYL (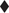) and sugarcane bagasse –SCB (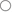). **(B)** Total amount of reducing sugars (mg/ml) produced by the consortium on XYL and SCB along a batch of culture (12–192 h). Relative enzymatic activities (β-xylosidase: 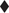 pNPβXyl; α-glucosidase: 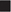; pNPαGlu; β-galactosidase: 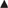 pNPβGal; and α-mannosidases: 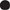 pNPαMan) of the consortium along a batch of culture on **(C)** XYL and **(D)** SCB. All the analyses were performed using three biological replicates and the error bars correspond to standard deviations.

The growth of the consortium was evaluated on four carbon sources with different structural complexity. The results showed that the consortium is able to grow using GLC, XYL, and SCB, but not CMC as a sole source of carbon (**Figure 1A**). In the GLC culture, a maximal growth was observed at 48 h, gradually decreasing toward the end of the batch (192 h). Regarding the XYL culture, we observed the exponential growth phase after 12 h, followed by the start of the stationary phase at 96 h. In the case of SCB, the exponential phase started at an early growth stage (between 0 to 12 h), and apparently was maintained until 96 h of the culture. Subsequent analyses were performed on the cultures with bacterial growth in substrates with higher complexity (XYL and SCB cultures).

### Quantification of Extracellular Sugars and Enzymatic Activities

In the XYL and SCB cultures, the concentration of the total proteins secreted by the consortium was less than 0.1 mg/ml. Regarding release of reducing sugars, maximum values (0.30–0.34 mg/ml) for the XYL culture were found between 48 and 192 h, during the exponential and stationary phases. In the SCB, we observed an increase of sugars during the entire growth period, with a maximum value at the end of the culture (0.28 ± 0.01 mg/ml; mean ± SD) (**Figure 1B**). Along the incubation time, we evaluated four types of enzymatic activities (target enzymes that can act on terminal ends of substrates) related with hemicellulose (β-xylosidase, β-galactosidases, α-mannosidase) and pectin (α-glucosidase) degradation processes. Thus, the consortium cultivated on XYL displayed maximal β-xylosidase and α-glucosidase activity values at 48 and 192 h, which was consistent with the sugar accumulation data (**Figure 1C**). Concerning the consortium cultivated on SCB, we observed a high β-xylosidase, α-glucosidase and β-galactosidases activity at 48 h versus a low α-mannosidase activity. The activities were relatively stable until the last measurement, except for β-xylosidase where a significant peak (*p* < 0,001, *t*-test of pairwise comparisons) was observed at 48 h (**Figure 1D**).

### Metatranscriptomic Overview of the Synthetic Bacterial Consortium Cultivated on Xylan and Sugarcane Bagasse

Total RNA extraction from the consortium cultivated on XYL and SCB was performed on triplicate systems along four time points of incubation. However, it was not possible to isolate sufficient RNA at the initial stage (12 h) of the XYL culture due to the low microbial activity and the presence of undigested xylan. Thus, a total of 21 samples were subjected to mRNA sequencing (9 from XYL and 12 from SCB cultures). Approximately 5.7 and 7.1 Gb of total mRNA sequencing data were obtained for the XYL and SCB samples, respectively. The approximately 116 million reads were assembled into 59,734 contigs. Given that N50 values discard read coverage information, we report the E90N50 value (1.1 kb) as a more useful indicator of transcriptome assembly quality than the N50 (665 bp) (Haas et al., 2013). Also note that the E90 number of transcripts (23,364) for which the E90N50 value is computed is roughly a third of the total number of transcripts assembled (68,738) and for which the N50 statistic was calculated. Based on the RNA-Seq fragment raw counts, we observed that 4,213 transcripts were differentially expressed in all pairwise combinations. As expected, the clustering of these transcripts showed that the XYL and SCB cultures had different expression profiles. For each, the profiles clustered by triplicates divided by treatment. Only the SCB time points 96 and 192 h formed an intermingled cluster based on the differential expression profile (**Supplementary Figure S2**), suggesting that after 96 h of growth on SCB the consortial expression patterns were ‘stable.’

### Relative Expression Profile of Plant Biomass-Degrading Enzymes in the Synthetic Bacterial Consortium Cultivated on Sugarcane Bagasse

**FIGURE 2:**
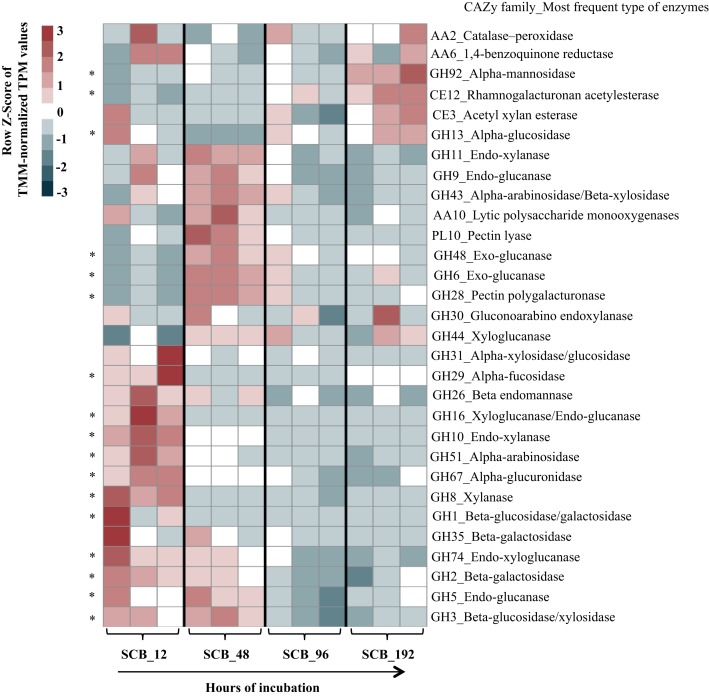
Heat map displaying the relative expression values (row *z*-score of TMM-normalized TPM) of thirty CAZy families (mostly involved in plant biomass degradation) in the synthetic bacterial consortium along a batch of culture (12–192 h) on sugarcane bagasse (SCB). Asterisks represent families differentially segregated along the incubation time that were identified by Boruta featured selection.

**FIGURE 3:**
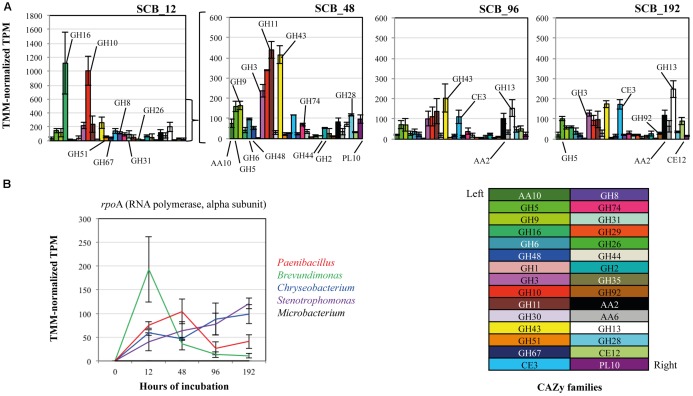
**(A)** Relative expression values (TMM-normalized TPM) of thirty CAZy families in the synthetic bacterial consortium, cultivated on sugarcane bagasse (SCB), within each incubation time. Error bars correspond to standard deviations. **(B)** Relative expression values and taxonomic affiliation of *rpo*A gene transcripts (RNA polymerase alpha subunit; KEGG orthology number K03040) within the consortium members along a batch of culture. In time zero, we assumed a low metabolic activity and probably undetected expression of *rpo*A gene.

Thirty CAZy families were selected in order to evaluate the expression of transcripts related with PB-degrading enzymes in the consortium. Here, we focus our analyses on the SCB cultures. A heat map using TMM-normalized TPM values showed a trend in the expression profiles of enzymes from the consortium along the incubation time (**Figure 2** and **Supplementary Table S1**). In general terms, most of the selected CAZy families were highly expressed at 12 and 48 h compared with 96–192 h. For instance, xyloglucanases/endo-glucanases (GH16), endo-xylanases (families GH8 and GH10) and α-glucuronidases (GH67) were significantly expressed at 12 hrs. Moreover, lytic polysaccharide monooxygenases -LPMOs (AA10), exo-glucanases (GH6 and GH48), α-arabinosidase/β-xylosidase (GH43), next to other endo-glucanases (GH5 and GH9) and endo-xylanases (GH11) were highly expressed at 48 h compared with all other time points. In addition, enzyme families involved in pectin degradation (e.g., GH28 and PL10) and β-glucosidases (specifically GH3) were mostly expressed at 48 hrs. Regarding lignin degradation, the family AA2 (catalases-peroxidases) showed higher values of expression at 12 (117 ± 42) and 192 hrs (118 ± 26). In terms of relative expression values (i.e., TMM-normalized TPM) between the CAZy families in each time point, we observed that transcripts of enzymes of families GH16 (1115 ± 447) and GH10 (1005 ± 207) were highly expressed at 12 h (**Figure 3A**). Moreover, those from families GH11 (439 ± 42), GH10 (339 ± 2.5), GH43 (414 ± 44), GH3 (236 ± 31) and GH9 (163 ± 20) were the most expressed at 48 hrs. At the final stages of the cultures (96–192 h), the relative expression values of enzymes belonging to families GH43, GH13, GH11, GH3 and CE3 were higher in comparison with those of the other families.

### Taxonomic Affiliation of Expressed Transcripts Related with Thirty Plant Biomass-Degrading Enzymes in the Synthetic Bacterial Consortium

**FIGURE 4:**
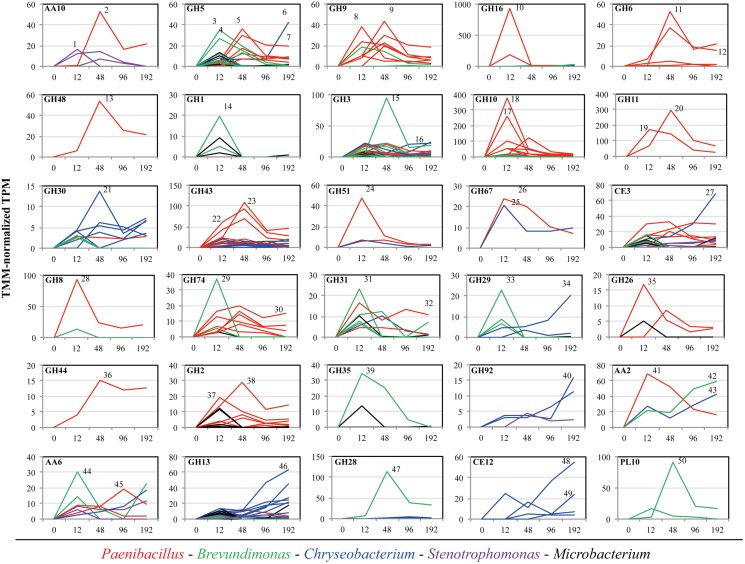
Taxonomic affiliation and TMM-normalized TPM values (average) of individual transcripts within thirty CAZy families. Axis *X* shows the incubation time of the synthetic microbial consortium cultivated on sugarcane bagasse (SCB). In time zero, we assumed a low metabolic activity and probably undetected expression of these CAZy families. Average transcription values lines affiliated to *Paenibacillus*, *Brevundimonas*, *Chryseobacterium*, *Stenotrophomonas*, and *Microbacterium* are colored in red, green, blue, purple and black, respectively. Numbers beside the highest expression lines within each panel correspond to the transcript ID in the **Supplementary Table S2**.

The relative expression of the *rpo*A gene (housekeeping single-copy gene throughout bacteria) was used as a marker to evaluate the metabolic activity of each member the consortium along the incubation time on SCB, thus providing a depiction of the relative contribution of each strain in the CAZy expression profile described above. The taxonomic affiliation and the TMM-normalized TPM values of the *rpo*A transcripts showed that *Brevundimonas* and *Paenibacillus* were the most active members at 12 and 48 h, respectively. In contrast, *Chryseobacterium* and *Stenotrophomonas* were highly active at 96–192 h compared with the other three consortium members. *Microbacterium* showed low activity along the entire incubation time (**Figure 3B**). In addition, we characterized all transcripts encoding enzymes of the thirty CAZy families to species (**Figure 4**). This analysis indicated that *Brevundimonas* and *Paenibacillus* were major contributors to the expression of enzymes involved in cellulose degradation. Specifically, *Brevundimonas* was relevant in the expression of β-glucosidases/galactosidases (GH1 and GH3) at 12 and 48 h (transcript IDs 14 and 15). The expression of enzymes of CAZy families GH10, GH11, GH30, GH43, GH51, and GH67, *Paenibacillus* and *Chryseobacterium* were the most important drivers of the degradation of xylan/arabinoxylan along incubation time (transcript IDs 17–26). In addition, endo-xylanases (GH8), endo-xyloglucanases (GH74), β-galactosidases (GH2), xyloglucanases/mannosidases (GH44) and β-endomannases (GH26) were mainly expressed by *Paenibacillus* at 48 h. Pectin degradation was mainly associated with the expression of enzymes of families GH28 and PL10 from *Brevundimonas* at 48 h (transcript IDs 47 and 50). The expression of catalases-peroxidases (AA2) was carried out by *Paenibacillus* (at 12 h), *Chryseobacterium* and *Brevundimonas* (at 192 h). Taxonomic affiliation, TMM-normalized TPM values (average and standard deviations) and the functional annotation of the 50 most important transcripts within the 30 CAZy families can be found in the **Supplementary Table S2**.

## Discussion

**FIGURE 5:**
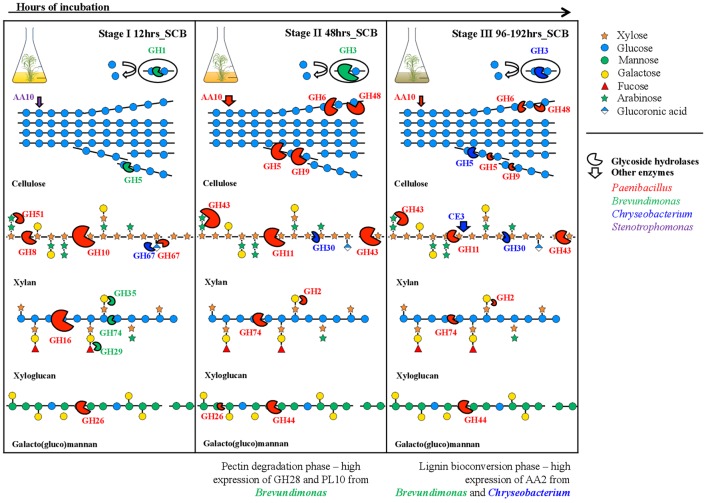
Cartoon of the successional profile representing the expression of lignocellulolytic enzymes by the synthetic bacterial consortium, along a batch of culture on sugarcane bagasse (SCB), in relation to the degradation process in the (hemi)cellulosic fraction of the plant biomass. The model shows how the selected CAZy families can degrade hemicellulose based on the reported evidence and also the taxonomic affiliation to genus level of the proteins expressed by members of the consortium. The size of the enzymes (semi-circles shape) is relative proportional to the relative expression values. Enzyme shapes colored in red, green, blue and purple correspond to enzymes expressed mostly by *Paenibacillus*, *Brevundimonas*, *Chryseobacterium*, and *Stenotrophomonas*, respectively. Specific molecules conforming each polymer follow the shape and color code shown in the top right corner.

In this study, we applied a metatranscriptomics-based approach to unveil the temporal expression dynamics of thirty PB-degrading enzymes (or CAZy families) in a synthetic consortium composed of five bacterial strains that was previously selected for its high degradation capacity. Overall, the data suggest that a specific successional expression profile of lignocellulolytic enzymes occurred in the consortium cultivated on SCB. For instance, the expression of specific endo-glucanases and endo-xylanases becomes highly relevant at 12–48 h, whereas enzymes acting on the external side linkages of the hemicellulose (e.g., α-arabinosidases/β-xylosidases) and cellulose (exo-glucanases and β-glucosidases) fractions are relatively highly expressed only after 48 h of cultivation (**Figures 2**, **5**). It is important to highlight that all predicted functions for the CAZy families were based on sequence annotation and that we observed high variability in the expression of some CAZy families, indicating that the different flasks (biological replicates) might show slight variation in the relative abundance of each strain. Nevertheless, the observed profile fits a conceptual model that was recently proposed (Jiménez et al., 2017) and suggests the successive stages of PB deconstruction. The main findings of this study are discussed based on three stages: 12, 48, and 96–192 h.

### First Stage (12 h)

Out of the thirty CAZy families evaluated, a total of 23 were highly expressed at this stage (row *Z*-score > 0 in at least one biological replicate). By using a random forest analysis and the Boruta statistical test, eleven CAZy families were significantly more expressed at 12 h (**Figure 2**), suggesting that this is the most functionally diverse phase compared with the other time points. In this stage, transcripts encoding enzymes of CAZy families GH8, GH10 and GH16 were significantly expressed compared with other time points. The family GH16 contains enzymes that can be assigned to five subgroups according to their substrate specificities, including xyloglucan transglucosylases/hydrolases (XTHs), (1,3)-β-galactanases, (1,4)-β-galactanases/κ-carrageenases, “non-specific” (1,3/1,3; 1,4)-β-D-glucan endohydrolases and (1,3; 1,4)-β-D-glucan endohydrolases (Strohmeier et al., 2004). Specifically, in our study, the high expression of this family at 12 h was due to one transcript that was affiliated to a 1,3-1,4-endoglucanase from *Paenibacillus* (78% identity) (**Supplementary Table S2**). The relevance of GH16 proteins has been acknowledged in previous studies. For instance, proteins from this family have been found in high relative abundances in the metagenomes of three aerobic PB-degrading microbial consortia (Jiménez et al., 2016). In addition, Zhou et al. (2014) reported that one of the microbial members (*Clostridium -*FC2) of the cellulolytic consortium F1RT has the capacity to produce cellulosomal enzymes belonging to family GH16. In *Trichoderma reesei* and *T. harzianum* these types of enzymes have been found highly expressed (or induced) in presence of cellulosic substrates (e.g., cellulose, avicel and sophorose) (Häkkinen et al., 2012; Ferreira Filho et al., 2017). On the other hand, proteins from GH16 have a putative structural and evolutionary connection with endoxylanases from family GH11 (Strohmeier et al., 2004). Based on the lack of experimental support on the cellulolytic activity of GH16 transcripts, we can speculate that, in our system, these enzymes may have an important role in transformation of hemicellulose (e.g., xyloglucan) present in the SCB. Based on the ability of our consortium to grow on GLC, XYL and SCB, but not on CMC, we suggest that the expression of enzymes involved in cellulose degradation (e.g., endo-glucanases) could be regulated by the presence natural cellulose of other type of PB polysaccharides present in the SCB. CMC could be used as an inductor of endoglucanases (Xiao et al., 2005). However, this polymer has a different structure compared to the natural cellulose found in PB.

In our study, two transcripts (IDs 17–18) contributed to a large extent to the high expression level of enzymes from family GH10 at 12 h (**Figure 4**). These were affiliated to endo-1,4-β-xylanases from *Paenibacillus* (>96% identity). Proteins from the family GH10 and GH16 were also strongly expressed and secreted in a corn stover-adapted microbial consortium at 24 and 72 h, respectively (Zhu et al., 2016). The high expression level of endo-xylanases in the consortium at 12 h suggests that xylan/arabinoxylan is attacked at early growth stages. One of the most important features of this stage was the high metabolic activity of *Brevundimonas* (**Figure 3B**). This organism may participate in the expression of endo-glucanases and endo-xyloglucanases from families GH5 and GH74, respectively. It is key to note that proteins from family GH5 can breakdown different plant polymers [e.g., cellulose, xyloglucan, and galacto(gluco)mannan]. Proteins derived from organisms affiliated with *Brevundimonas* species have been found in high relative abundance in two consortia cultivated with once-used PB (Jiménez et al., 2016). These results suggest that *Brevundimonas* may play an important role in xylan, cellulose and lignin degradation. Moreover, the expression of α-glucuronidases – which cleave 4-*O*-methyl-α-D-glucuronic acid (MeGlcA) from xylose residues when they are attached to the terminal, non-reducing end of xylo-oligosaccharides (Dodd and Cann, 2009) – by *Chryseobacterium* and *Paenibacillus* was significantly higher at 12 h, indicating that the consortium can carry out this process at the early stages of cultivation. Interestingly, the removal of MeGlcA could expose the xylan to attack by endo-xylanases and β-xylosidases (Rogowski et al., 2014). Two transcripts (ID 41 and 44) affiliated to AA2 and AA6 (and derived from *Paenibacillus* and *Brevundimonas*, respectively) were comparatively highly expressed at 12 h (**Figure 4**). We speculate that the expression of these transcripts is probably related to cell protection from oxidative damage and the transfer of electrons.

At this stage, the low cell densities and the presence of accessible PB polysaccharides could enhance the fast growth of *Paenibacillus* and *Brevundimonas*. Here, competition and niche partitioning events are likely to occur (Jiménez et al., 2017). Thus, based on the high expression of *Brevundimonas*-derived transcripts affiliated to α-fucosidases (GH29) and β-galactosidases (GH35) at this stage (**Figure 4**), we suggest that this species may consume preferably fucose and galactose, whereas *Paenibacillus* could have more access to arabinose and mannose. Competition for the most abundant sugars (glucose and xylose) could be a valid explanation for the lower activity of *Brevundimonas* in the next stages. At an early phase, *Microbacterium* could consume easily available oligo- or monosaccharides present in the SCB. However, we suggest that the low metabolic activity of *Microbacterium* could be directly related with an intrinsic characteristic of the organism (e.g., oligotrophy), niche competition and possibly a low metabolic versatility. Probably, in the consortium, *Microbacterium* could confer functional stability, redundancy and robustness, thus increasing the metabolic diversity and providing a potential buffer against external disturbance (Awasthi et al., 2014). In addition, *Microbacterium* species could secrete molecules that synergistically stimulate the degradation capacity of other PB-degrading bacterial species.

### Second Stage (48 h)

This is the key stage for the degradation of the main PB polysaccharides. Indeed, we observed raised extracellular enzymatic activities at 48 h, with a significant peak of β-xylosidase (**Figure 1D**). Here, efficient hydrolysis of cellulose can be carried out by a synergistic action of non-processive endo-glucanases (GH5 and GH9), which produce new ends at random within the polysaccharide chain, and processive exo-glucanases (GH6 and GH48), which remain attached to the substrate and split off cellobiose from such free ends. Then, cellobiose can be converted to glucose by the action of β-glucosidases expressed by *Brevundimonas*. An interesting synergistic interaction is carried out at this stage, where consumption of cellobiose by *Brevundimonas* potentially prevents metabolite repression of endo/exo-glucanases produced by *Paenibacillus* (Lynd et al., 2002; Berlemont and Martiny, 2013). Moreover, LPMOs from *Paenibacillus* may play a relevant role in the cellulose degradation at this stage. These LPMOs are metalloenzymes (copper-dependent) that catalyze the oxidative cleavage of (1,4)-linked glycosidic bonds of crystalline PB polysaccharide surfaces (Horn et al., 2012; Agger et al., 2014).

Concerning hemicellulose degradation, we found that transcripts affiliated to *Paenibacillus* from the families GH11 (endo-xylanases) and GH43 (α-arabinosidases/β-xylosidases) were highly expressed when compared to the other time points. We found a shift in the expression of endo-xylanases – i.e., from GH10 at 12 h to GH11 at 48 h – which display an endo-action and thus hydrolyze β-(1,4)-linkages between adjacent xylose residues within the xylan/arabinoxylan chain. The enzymes of these families essentially differ in their structure and substrate specificity. For instance, GH10 xylanases have a (β/α)_8_-fold and are much more versatile. In contrast, GH11 enzymes are the smallest xylanases with a β-jelly-roll architecture, are highly substrate-specific and do not tolerate high substitutions on the backbone (Biely et al., 1997; Paës et al., 2012). This suggests that a high level of enzymatic complementarity can occur in *Paenibacillus* within the consortium along the growth period. Regarding GH43, it can display dual action (Maruthamuthu et al., 2017), thus removing arabinose from the external-side chains attached to the xylan backbone or hydrolyze xylobiose into its monomeric units, releasing D-xylose (Rytioja et al., 2014). The family GH43 enzymes seem to play a key role in lignocellulose degradation, being highly enriched in two soil-derived microbial consortia cultivated on wheat straw (Jiménez et al., 2015a) and highly expressed and secreted between 72 to 168 h in PB-degrading microbial consortia cultivated under aerobic and anaerobic conditions (Abot et al., 2016; Zhu et al., 2016).

Although *Paenibacillus* was the most metabolically active organism and possibly the biggest contributor to the (hemi)cellulose deconstruction, *Brevundimonas* may play a key complementary role through the expression of pectinolytic enzymes (e.g., GH28 and PL10). Pectins comprise a diverse and complex group of polymers that function as structural components in PB (Mohnen, 2008). Thus, the action of pectinolityc enzymes could increase the sugar release from the (hemi)cellulosic fraction (Xiao and Anderson, 2013). Theoretically, different consortium members can contribute in distinctive ways and proportions to the SCB degradation process. However, we suggest that organisms that contribute more to the substrate transformation may profit from the most abundant sugars (glucose or xylose) (Jiménez et al., 2017). Based on this conceptualization, at this stage, *Paenibacillus* might indeed be the most abundant organism in terms of cell density. In other words, *Paenibacillus* invests a lot in the production of lignocellulolytic enzymes, and it might grow very well at the expense of the large proportion of sugars.

### Third Stage (96–192 h)

At this final stage, the expression of the majority of the CAZy families decreased. A similar trend occurs with the metabolic activity of *Paenibacillus* and *Brevundimonas*. These two organisms may be acting as biggest contributors to the degradation process at early stages (12 and 48 h) of the culture, At this stage, *Paenibacillus* probably entered stationary phase, which is reflected in the decrease of GHs gene expression. We suggest that at these time points the major fraction of the available (hemi)cellulose is already degraded. Here, not only carbon sources but also other essential nutrients, such as nitrogen, can become progressively limiting. Although production of sugars (by PB polysaccharide degradation) and their consumption occurs simultaneously in the consortium, we observed an increase in the concentration of reducing sugars along the time of incubation (**Figure 1B**). This suggests that, at the end of the culture, the environment becomes relatively more homogeneous. Thus, the increasing release of easily assimilable carbon sources is expected to enhance competition among the bacterial strains. *Stenotrophomonas* and *Chryseobacterium* showed higher normalized expression values of their *rpo*A gene at the end of the culture (192 h) (**Figure 3B**), suggesting that they become highly metabolically active when the concentration of monosaccharides is higher. We considered *Stenotrophomonas* to be a ‘sugar cheater’ in the consortium. That means that it profits from the sugars made available by the other community members, without effectively contributing to the PB degradation process (Jiménez et al., 2017). This is the case of *Thermus thermophilus*, which lacks the metabolic potential for PB polysaccharide hydrolysis, in a switchgrass-degrading consortium (D’haeseleer et al., 2013). Moreover, the exact functional roles of *Microbacterium* and *Stenotrophomonas* in the consortia remained unknown and further studies need to be performed in order to understand their impact in this system. However, we speculate that they could be involved in feedback regulation, cross-feeding, maintenance of functional stability and inhibition of metabolite repression. Finally, at this stage, lignin bioconversion was thought to take place by the expression of catalases-peroxidases (CAZy family AA2) from *Chryseobacterium* and *Brevundimonas.* Similar to patterns observed in composting (Antunes et al., 2016), where high expression of ligninolytic enzymes occurs at the end of the process.

## Conclusion and Final Remarks

In this study, we provide evidence for a temporally explicit dynamics of expression of PB-degrading enzymes in a five-species synthetic bacterial consortium growing on a complex substrate such as SCB. Initially, *Paenibacillus* and *Brevundimonas* were found to become active, presumably synergistically, in the degradation of cellulose and hemicellulose (between 12 to 48 h), whereas *Chryseobacterium* and *Stenotrophomonas* were most active at the end of the culture. Based on our results, we suggest that the consortium may follow an a ‘endogenous heterotrophic succession’ trajectory (Fierer et al., 2010; Jiménez et al., 2017), which is characterized by a rapid initial activity of so-called pioneer populations that initiate the deconstruction of PB (in the consortium, *Paenibacillus* and *Brevundimonas*), followed by the relative decline of these initial populations. Other species that outcompete the pioneer populations in sugar uptake then start to proliferate (in the consortium, *Chryseobacterium* and *Stenotrophomonas*). Thus, we posit that a shift from synergism to competition is an important trait in these systems. This dichotomy could be correlated with the complexity of the available carbon sources (Deng and Wang, 2016).

Understanding the ecological interactions and the temporal expression dynamics of PB-degrading enzymes in a synthetic microbial consortium can be used to improve aerobic saccharification processes for biofuel production. Thus, we propose a dynamic saccharification process where two types of enzyme cocktails could be applied in a sequential manner. The first cocktail could contain enzymes acting mainly in the backbone of hemicellulose (e.g., xyloglucanases/endo-glucanases from CAZy family GH16; and endo-xylanases from families GH8 and GH10). The second cocktail could be composed by a diverse array of endo-enzymes, exo-enzymes and LPMOs where proteins from CAZy families GH1, GH3 (β-glucosidases/galactosidases), GH5, GH9 (endo-glucanases), GH6, GH48 (exo-glucanases), GH11, GH30 (endo-xylanases) and GH43 (α-arabinosidase/β-xylosidases) need to be considered as essential components. Although lignin is an important polymer of PB, it is still unclear which type of lignolytic enzymes could increase the release of sugars from the (hemi)cellulosic fraction. This last subject will require more attention in the future.

As a cautionary note, mRNA abundance is not necessarily indicative of protein levels and enzymatic activities, and so it is possible that actual activities last longer than the time of (instantaneous) mRNA detection. One may argue here that the successional expression dynamics of PB-degrading enzymes on the basis of mRNA provides an overview of the inferred activity dynamics, and so still improves the ecological understanding of PB-degrading microbial consortia. Metaproteomics approaches as well as the relative abundances (e.g., cell population densities and/or 16S rRNA gene copy numbers) of each organism – growing alone and in a consortium – could improve our understanding of the successional dynamics in these systems. In addition, the growth of the individual strains on different carbon sources would unveil substrate preferences, interactions, and niche partitioning and competition events. Moreover, we suggest that further enzymatic tests, substrate composition and chromatographic analysis could improve the evidence that the bacterial consortium starts breaking the internal bonds of the plant polysaccharides, moving to the external branches as succession takes place.

## Author Contributions

DJJ performed most of the experiments, participated in the bioinformatic analyses and drafted the manuscript. MCDM performed all the bioinformatic procedures. JFS conceived the study, participated in its design, coordination, and contributed to the drafting of the manuscript.

## Conflict of Interest Statement

The authors declare that the research was conducted in the absence of any commercial or financial relationships that could be construed as a potential conflict of interest.

## Supplementary Material

**FIGURE S1 |** Flasks containing the synthetic bacterial consortium at 96 h of growth in **(A)** glucose -GLC, **(B)** carboxymethylcellulose -CMC, **(C)** xylan -XYL and **(D)** sugarcane bagasse –SCB. Flasks marked with WI are without inoculum.

**FIGURE S2 |** Heat map showing the expression values (Log2_TMM) of all significantly differentially expressed transcripts of the synthetic bacterial consortium across all samples along the batch of culture in xylan –XYL (48, 96, and 192 h) and sugarcane bagasse –SCB (12, 48, 96, and 192 h).

**TABLE S1 |** Relative expression values (TMM-normalized TPM) used to construct the heat map in the **Figure 2**.

**TABLE S2 |** Taxonomic affiliation, TMM-normalized TPM values (average and standard deviations-SD) and the functional annotation (by BLASTp) of the 50 most important transcripts, pointed out in the **Figure 4**, within the thirty CAZy families.
