# Supplementary material for: Temporal Expression Dynamics of Plant Biomass-Degrading Enzymes by a Synthetic Bacterial Consortium Growing on Sugarcane Bagasse
Source: Front Microbiol. 2018 Feb 26;9:299. doi: 10.3389/fmicb.2018.00299 (PMC5834485; doi:10.3389/fmicb.2018.00299)
Supplement: Supplementary file 1 [file Presentation_1.PPT]

## Slide 1
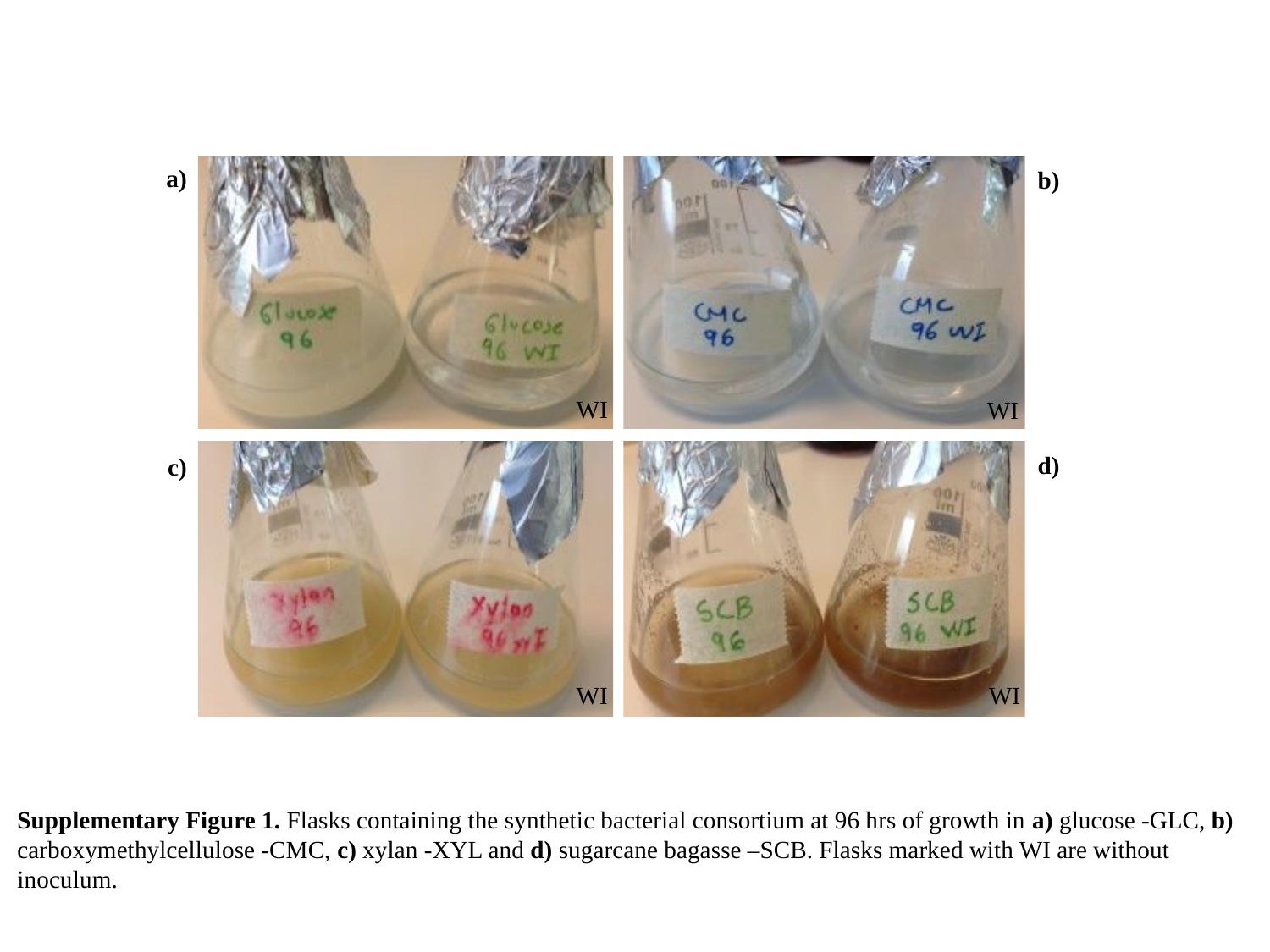

a)
b)
WI
WI
d)
c)
WI
WI
Supplementary Figure 1. Flasks containing the synthetic bacterial consortium at 96 hrs of growth in a) glucose -GLC, b) carboxymethylcellulose -CMC, c) xylan -XYL and d) sugarcane bagasse –SCB. Flasks marked with WI are without inoculum.

## Slide 2
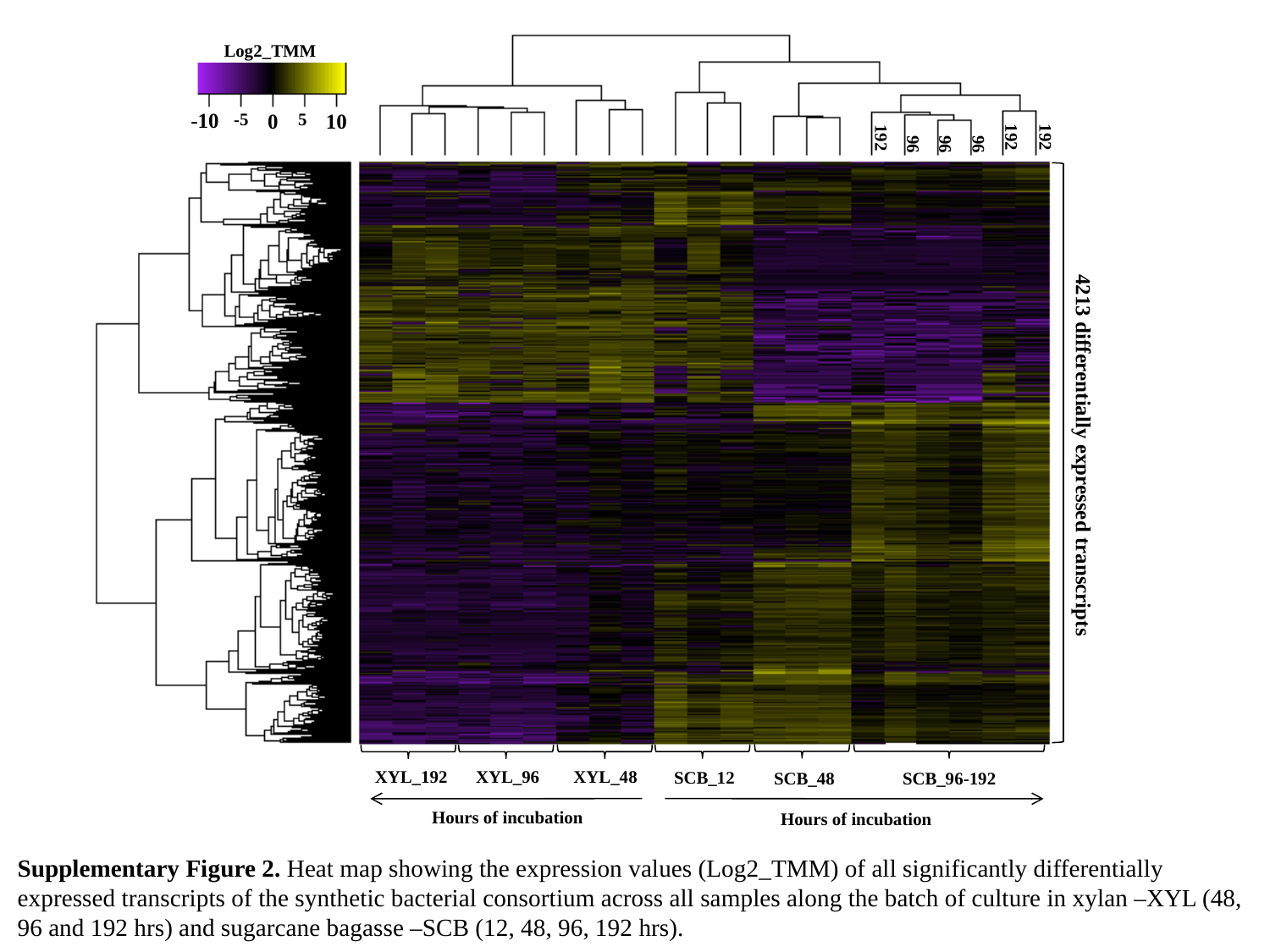

Log2_TMM
-10
0
5
-5
10
192
192
192
96
96
96
4213 differentially expressed transcripts
XYL_192
XYL_96
XYL_48
SCB_12
SCB_48
SCB_96-192
Hours of incubation
Hours of incubation
Supplementary Figure 2. Heat map showing the expression values (Log2_TMM) of all significantly differentially expressed transcripts of the synthetic bacterial consortium across all samples along the batch of culture in xylan –XYL (48, 96 and 192 hrs) and sugarcane bagasse –SCB (12, 48, 96, 192 hrs).
